# Delivering PrEP to Young Women in a Low-Income Setting in South Africa: Lessons for Providing Both Convenience and Support

**DOI:** 10.1007/s10461-021-03366-x

**Published:** 2021-07-14

**Authors:** Tali Cassidy, Nelisiwe Ntuli, Charllen Kilani, Nikiwe Malabi, Bulelwa Rorwana, Tabitha Mutseyekwa, Rebecca O’Connell, Sarah Jane Steele, Zee Ndlovu, Tom Ellman, Virginia de Azevedo, Colin Pfaff, Aurelie Nelson, Laura Trivino Duran

**Affiliations:** 1 Médecins Sans Frontières, Cape Town, South Africa; 2 Division of Public Health Medicine, School of Public Health and Family Medicine, University of Cape Town, Cape Town, South Africa; 3 Southern African Medical Unit, Cape Town, South Africa; 4 City Health Department, Cape Town, South Africa

**Keywords:** Pre-exposure prophylaxis, Young women, HIV prevention, Africa, Adherence

## Abstract

Daily oral pre-exposure prophylaxis (PrEP) is a key tool in addressing high HIV incidence among young women, and breaking the cycle of transmission. From 2017 to 2020, Médecins Sans Frontières (MSF) offered PrEP, in conjunction with contraception and risk-reduction counselling, to women aged 18–25, in a government-run clinic in Khayelitsha, a low income high HIV prevalence area in South Africa. Drawing on clinical, quantitative, and qualitative interview data, we describe participants’ experiences and engagement with the PrEP program, participant adherence (measured by TFV-DP levels in dried blood spots) over time, and the indirect benefits of the PrEP program. Of 224 screened and eligible participants, 164 (73.2%) initiated PrEP, with no large differences between those who initiated and those who did not. Overall, 47 (29%) completed 18 months follow-up, with 15 (9.1%) attending all visits. 76 (46.9%) participants were lost to follow-up, 15 (9.1%) exited when leaving the area, and 28.7% of exits happened in the first month of the study. We identified two different trajectories of PrEP adherence: 67% of participants had, on average, consistently low TFV-DP levels, with the remaining 33% having sustained high adherence. Few baseline characteristics predicted good adherence. The main reported barrier to taking PrEP was forgetting to take or travel with the pills. Encouragement from others declined as a reported facilitator from month 6 to 18 (family: 93.1% vs 77.6%, p = 0.016, friends: 77.6% vs 41.4%, p ≤ 0.001, partners: 62.1% vs 46.6%, p = 0.096, other PrEP users: 89.7% vs 74.1%, p = 0.020). Disclosure to friends and family in some cases opened dialogue around sex, and helped to educate others about PrEP. Self-reported sex with more than one partner, and sex without a condom, decreased significantly after enrolment (p < 0.001, p = 0.063). In the individual interviews, participants credited their PrEP experience with changing their behaviour. Recognising the challenges with, but overall benefits from a package of care that includes the option of PrEP, lessons drawn from this study can help maximise persistence on PrEP within resource constraints. PrEP providers need to address participants’ need for both convenience and social support.

**Supplementary Information:**

The online version contains supplementary material available at 10.1007/s10461-021-03366-x.

## Introduction

In South Africa 7.7 million people are living with HIV [1], constituting 20% of the global HIV-positive population [2]. Among youth aged 15–24 years, 88,000 new infections occur annually, translating to an annual incidence of 0.49% among males, and 1.51% among females [3]. Similar trends are reflected throughout Southern and East Africa: adolescent girls and young women (AGYW) made up 72% of the 262,000 new HIV infections in this age group in 2019 [4]. HIV prevention in AGYW is key to address the high incidence in this group, and to break the cycle of transmission [5, 6].

One potential safe and efficacious strategy for HIV prevention is daily oral pre-exposure prophylaxis (PrEP) [7], consisting of tenofovir (TFV) and emtricitabine. PrEP efficacy was first demonstrated among men who have sex with men (MSM) [8–10] and has also been shown to reduce incidence in injecting drug users [11]. Efficacy among heterosexual women, while lower, has been demonstrated in trials [12, 13] and open label pilots [14, 15] in southern and east Africa. However, trials in this population have had mixed results, with the VOICE trial in South Africa, Uganda and Zimbabwe [16] and FEM-PrEP in Kenya, South Africa and Tanzania [17], showing no efficacy. This is likely due to low levels of measured adherence found in these studies.

Implementation of PrEP has also been hindered by inconsistent visit attendance and low adherence, measured in a variety of ways. Studies of integration of PrEP into routine services in Kenya have shown low uptake of PrEP [18], and low proportions (41%) returning one month after initiation [19, 20]. An analysis of over 40,000 PrEP clients in sub-Saharan African countries showed that disengagement (and sometimes re-engagement) is common [21]. Less than half of women (aged 15–19) had detectable plasma TFV levels in a South African setting [22]. A trial in South Africa, Uganda, and Zimbabwe showed 50% TFV detection in quarterly plasma samples in AGYW [16]. In month 3 of the HPTN 082 trial, 25% of young women in South Africa and Zimbabwe had tenofovir diphosphate (TFV-DP) levels consistent with taking four or more pills/week [23]. This figure was 44% in a separate study of female sexual partners of migrant miners in Mozambique [24]. In rural Kenya and Uganda 27% of young women had detectable TFV in hair samples [25].

Despite these challenges, data suggest that PrEP can reduce HIV incidence outside of a trial setting. In the SEARCH study, in rural Uganda and Kenya, after PrEP became more widely available, there was 79% lower HIV incidence among PrEP initiators with follow-up HIV testing than among matched controls from before PrEP availability [26]. In the ECHO study HIV risk was reduced in the overall cohort after PrEP became available in 12 sites in four sub-Saharan African countries [27].

The emerging consensus is that PrEP programs for AGYW in Africa need to address several key challenges. Firstly, programs need to improve knowledge of PrEP among healthcare providers and women [28]. Secondly, programs also need to find ways to integrate PrEP with other HIV prevention and sexual and reproductive health (SRH) services, including counselling, STI screening and treatment [28] and family planning. In a Kenyan program, young women who initiated PrEP concurrently with contraception had slightly better one-month continuation rates (39.4%), than those initiating only PrEP (36.2%) [20], though these low rates suggest that this is only partially successful. Finally, previous experience of PrEP programs in Africa has highlighted the need to improve PrEP access by maximizing convenience, access, and support [29], and more strategies to ensure adherence and consistent access to PrEP are needed [28].

In this context, from 2017 to 2020, Médecins Sans Frontières (MSF) ran a pilot study of daily oral PrEP for women aged 18–25, in a government-run clinic in Khayelitsha, South Africa. PrEP was offered in conjunction with their contraception, as part of an SRH package of care including social support and risk reduction counselling. We describe this cohort of young women who chose to initiate PrEP in a primary health care setting, their engagement with the PrEP program, and adherence at multiple time points. We highlight the indirect SRH-related benefits of the PrEP program. In light of these results, we present lessons learned from this program regarding convenience and support, which can inform future PrEP rollout.

## Methods

### Setting

Khayelitsha is a low-income, high HIV prevalence peri-urban area in Cape Town, where about 45% of households live in informal structures. Khayelitsha is home to approximately 500,000 people, 44% of whom are under the age of 25 [30, 31].

Recruitment and study visits took place at a youth clinic, on the same premises as a larger community health center. The youth clinic is run by the City of Cape Town’s health department and provides HIV testing, ART initiation and dispensing, contraception, STI screening and diagnosis, and basic curative services, exclusively to youth aged 12–25 years [32].

### Recruitment and Eligibility

MSF employed a study nurse and counselor, who were based at the youth clinic and occasionally assisted with routine clinic patients. We requested that clinic staff refer any interested potential participants to the PrEP team, and distributed flyers, put up posters, and conducted health talks in the waiting rooms, to educate clinic attendees about PrEP. We enlisted the help of health promoters, at both our youth clinic and another youth clinic in Khayelitsha, to give PrEP talks and refer participants. Information about the study was also disseminated at MSF outreach events, and we made use of a local radio station to educate the community about PrEP and let prospective participants know about the study.

Prospective participants were consented, screened, and had bloods drawn at a screening visit. To be eligible to initiate PrEP at their next visit, consenting participants had to be between 18 and 25 years old, willing to use contraception (condoms included), Hepatitis B seronegative, HIV-negative, > 35 kg, have a creatinine clearance ≥ 60 mL/min, and not pregnant or breastfeeding. If participants reported a potential HIV exposure in the previous 72 h, they were initiated on post-exposure prophylaxis (PEP) immediately, and then, if eligible, continued onto PrEP when they completed their PEP.

### Intervention

After PrEP enrolment, participants returned for month 1 and 2 visits, and thereafter returned every two or three months, depending on their contraception schedule and preference. At each visit, routine medical checks were performed, including HIV and pregnancy testing and syndromic STI screening, and contraception was provided. No financial incentives were offered for enrolment, or any study visit attendance.

### Counseling and Peer Support

Risk-reduction counseling took place every visit in one-on-one sessions. Combining the principles of motivational interviewing [33] and “The Stages of Change” [34, 35], the counseling model covered (1) PrEP use (education, adverse events and continued effective use), (2) HIV risk assessment and reduction (identification of risk, risk reduction strategies and continued support), and (3) general SRH topics (sex and sexual health). Apart from adverse event management, handled by the clinical nurse practitioner only, both the nurse and the counselor were trained to provide the full counseling package [36].

Initially, we had aimed to have “SRH Clubs”, at each visit, where participants met in a group setting with a counsellor. However, due to a lack of participant availability or attendance, these were difficult to coordinate. After approximately three months of trying with no successful club sessions, we replaced SRH clubs with SRH “PrEP diva” gatherings, held on Saturdays. In total, seven events took place (approximately three/year). Similarly to clubs, these participant gatherings were a way of introducing participants to peers, and encouraging peer support through fun group discussions held outside the clinic setting led by study staff. We also had a WhatsApp group, which most participants opted to join, providing a convenient platform where participants could engage with study staff and each other.

### Quantitative Data Collection and Analysis

Baseline characteristics were recorded by the study nurse at the screening and enrolment visits. At each follow-up visit, routine medical data was recorded, and participants reported on their barriers and facilitators to PrEP use, perceived risk, and risk behaviours. On study exit, a form was completed documenting reasons for exit and overall experiences.

At month 1, 3 or 4 (depending on contraception schedule), 12, and 18, we measured blood levels of tenofovir diphosphate (TFV-DP) levels in dried blood spots (DBS). We used a cut-off of ≥ 700 fmol/punch to create a binary measure of good adherence (estimated ≥ 4 pills/week) [37], consistent with other studies [23, 24]. TFV-DP tests were processed in batches and results were not regularly available. Therefore, results were not fed back to participants.

We describe baseline participant characteristics, stratified by whether they successfully initiated PrEP. We divided the follow-up period into roughly 6-month intervals after enrolment: spanning visits at months 1–6, months 8/9–12, and months 14/15–18, with a window period of four weeks for those on the two-monthly schedule, and six weeks for those on the three-monthly schedule. In each time interval, we describe participants’ patterns of attendance, including missed visits, contraception schedule alignment and reasons for study exits. Participants were considered lost to follow-up if they did not return and were uncontactable for completion of the study exit form. For participants with at least one follow-up visit in each interval we describe the changing reported risk perception and behaviours, STI diagnoses, and reported barriers and facilitator to PrEP use over time, and examine trends using Kendall’s rank correlation test.

Latent groups of participants with similar PrEP adherence trajectories were identified using group-based trajectory modelling [38, 39]. Previous literature has suggested that early adherence predicts later adherence [16], and a recent study in a similar population identified two latent groups based on PrEP adherence trajectories [40]. We explored combinations of linear and quadratic models for two or three groups. A model was selected based on lowest Akaike Information Criteria (AIC), Bayesian Information Criteria (BIC) and log-likelihoods. For the purpose of this analysis, missing values of TFV-DP (where the participant did not attend the visit) were set to zero. This is in line with previous study [40], and based on the assumption that participants who did not attend the visit would have had low adherence levels around that point in time.

We use Cox proportional hazards regression to explore whether there were any baseline predictors of study exit (for any reason before completion), or loss to follow-up, with follow-up time starting at PrEP initiation. Similarly, binomial generalized linear models were used to explore associations between baseline characteristics and probability of successfully initiating PrEP, or being in the trajectory group with higher PrEP adherence.

Analyses were performed using Stata 15 [41].

### Qualitative Data Collection and Analysis

We recruited 14 study participants and nine friends and family of study participants for individual interviews. Participants were purposively sampled at different time points, and family and friends were contacted with participants’ consent. Qualitative data was transcribed and thematically analysed using Nvivo [42], adopting a grounded theory approach [43].

### Ethics

The University of Cape Town Human Research Ethics Committee and MSF’s ethics review board both granted ethics approval for this study (HREC 808/2016 and MSF #1647), and the qualitative substudy (HREC 99/2018 and MSF #1780). The study was also approved by the City of Cape Town’s health department (7747).

## Results

### Participant Characteristics

During the recruitment period, approximately 4916 women 18–25 years old not known to be HIV-positive attended Site C Youth Clinic for any services. Of 236 women who completed the full screening and consent visit, 12 (5.1%) were ineligible and of the 224 eligible, 164 (73.2%) returned to the clinic and successfully initiated PrEP, with a median time to initiation of 6 days (IQR 2–9). Study staff recruited 84 (35.6%) of the women, 70 (29.7%) were referred by a clinic counsellor, and 38 (16.1%) were referred by word of mouth. The median age of all recruited was 21 (IQR 19.6–22.9). Most women had a secondary/high school education level (n = 181, 76.7%), 104 (44.1%) were enrolled in a course or educational institution, 57 (24.1%) were employed (including 5.5% both working and studying), and 87 (36.9%) were neither employed nor studying. Overall 29 (12.3%) women had a syndromically diagnosed STI at baseline and 21 (8.9%) reported a recent HIV exposure and were initiated on PEP. There were no large differences between those who initiated PrEP and those who did not (Table 1).

**Table 1:** Baseline characteristics of study participants by PrEP initiation status

	Enrolled, PrEP not initiated	Initiated PrEP	Total
N	72	164	236
Reasons for not enrolling n (%)			
Eligible, did not return for enrolment	60 (83.3%)	–	–
HIV-positive	2 (2.8%)	–	–
Other medical reason	4 (5.6%)	–	–
Pregnant	1 (1.4%)	–	–
Eligible at screening but not enrolment*	5 (6.9%)	–	–
Median days between screening and enrolment (IQR)	–	6 (2–9)	–
Where/how did the participant hear about the PrEP study? n (%)			
Nurse	1 (1.4%)	1 (0.6%)	2 (0.8%)
Clinic counsellor	17 (23.6%)	53 (32.3%)	70 (29.7%)
Recruited by study staff	30 (41.7%)	54 (32.9%)	84 (35.6%)
Outreach outside of clinic	3 (4.2%)	3 (1.8%)	6 (2.5%)
Word of mouth	6 (8.3%)	32 (19.5%)	38 (16.1%)
Poster or flier in clinic	13 (18.1%)	16 (9.8%)	29 (12.3%)
Other	2 (2.8%)	5 (3%)	7 (3%)
Baseline characteristics at screening			
median age (IQR)	21.2 (20–23.4)	20.9 (19.5–22.7)	21 (19.6–22.9)
Highest education level passed, n (%)			
Primary school	14 (19.4%)	15 (9.1%)	29 (12.3%)
Secondary/high school	49 (68.1%)	132 (80.5%)	181 (76.7%)
Tertiary	9 (12.5%)	17 (10.4%)	26 (11%)
Employment status, n (%)			
Employed	12 (16.7%)	32 (19.5%)	44 (18.6%)
Studying	27 (37.5%)	64 (39%)	91 (38.6%)
Employed and studying	6 (8.3%)	7 (4.3%)	13 (5.5%)
None of the above	27 (37.5%)	60 (36.6%)	87 (36.9%)
Time living in Khayelitsha, n (%)			
Less than a year	4 (5.6%)	15 (9.1%)	19 (8.1%)
1–3 years	12 (16.7%)	16 (9.8%)	28 (11.9%)
Greater than 3 years	56 (77.8%)	133 (81.1%)	189 (80.1%)
Sexually active, n (%)	71 (98.6%)	161 (98.2%)	232 (98.3%)
STI diagnosed at enrollment, n (%)	10 (13.9%)	19 (11.6%)	29 (12.3%)
PEP started at enrollment (HIV exposure reported), n (%)	6 (8.3%)	15 (9.1%)	21 (8.9%)

*One pregnancy, one planned to move away, one had high creatinine, two had other clinical complications

### Patterns of Attendance: Scheduling, Contraception and Exits

Overall, 15 (9.1%) participants attended all visits, with 7 (4.3%) consistently attending within seven days of their scheduled date. By month 18, 25 (15.2%) had returned for a PrEP visit after three months with no PrEP. A large proportion (28.7%) of exits happened in the first month of the study. By month 18, 76 (46.9%) of participants were lost to follow-up and 47 (29%) completed their follow-up time. Aside from loss to follow-up and study completion, the mostly commonly cited reason for study exit was the participant leaving the area (9.1%). Of nine participants (5.5%) exiting because of side effects, six exited in the first month. There was only one pregnancy and one participant, who was likely to have been in the window period at PrEP initiation, seroconverted (Table 2).

**Table 2:** Patterns of attendance, timing of and reasons for exits, contraception alignment, and adherence measures, N = 164

	Month 0–1* n (%)	Month 2–6* n (%)	Month 8/9–12* n (%)	Month 14/15–18* n (%)	Month 0–18* n (%)
Attendance					
Attended all visits in period	110 (67.1%)	63 (38.4%)	47 (28.7%)	25 (15.2%)	15 (9.1%)
Attended all visits within 7 days of scheduled date in period	104 (63.4%)	42 (25.6%)	29 (17.7%)	15 (9.1%)	7 (4.3%)
Returned after > 3 months of no PrEP	–	7 (4.3%)	9 (5.5%)	10 (6.1%)	25 (15.2%)
Attended participant event in time period	8 (4.9%)	30 (18.3%)	31 (18.9%)	21 (12.8%)	54 (32.9%)
Still in study during period	–	117 (71.3%)	75 (51.8%)	68 (41.2%)	–
Exited** during period	47 (28.7%)	32 (19.5%)	19 (11.6%)	66 (40.2%)	164 (100%)
Reasons for exit					
Lost to follow-up	25 (53.2%)	21 (65.6%)	14 (77.8%)	17 (26.2%)	76 (46.9%)
Study complete	0 (0%)	0 (0%)	0 (0%)	46 (70.8%)	47 (29%)
HIV seroconversion	1 (2.1%)	0 (0%)	0 (0%)	0 (0%)	1 (.6%)
Pregnancy	0 (0%)	1 (3.1%)	0 (0%)	0 (0%)	1 (.6%)
Other clinical reasons	4 (8.5%)	1 (3.1%)	0 (0%)	1 (1.5%)	6 (3.7%)
Other reasons	17 (36.2%)	9 (28.1%)	4 (22.2%)	1 (1.5%)	31 (19.1%)
Other reasons (not mutually exclusive)					
Side effects	6 (12.8%)	2 (6.3%)	1 (5.6%)	0 (0%)	9 (5.5%)
Adherence problems	4 (8.5%)	2 (6.3%)	0 (0%)	0 (0%)	6 (3.7%)
No longer feels at risk of HIV	3 (6.4%)	2 (6.3%)	0 (0%)	0 (0%)	5 (3.0%)
Stigma/negative attitudes	2 (4.3%)	0 (0%)	0 (0%)	0 (0%)	2 (1.2%)
Leaving the area/travel	8 (17%)	4 (12.5%)	2 (11.1%)	1 (1.5%)	15 (9.1%)
Taking too much time	1 (2.1%)	0 (0%)	1 (5.6%)	0 (0%)	2 (1.2%)
Other	1 (2.1%)	1 (3.1%)	0 (0%)	1 (1.5%)	3 (1.9%)
Contraception alignment					
N on baseline injectable or pill and attended any visits in period	89	87	67	53	103
Received contraception (same type as baseline)	NA***	69 (79.3%)	41 (61.2%)	35 (66.0%)	85 (82.5%)
Received different contraception to baseline (not condoms)	NA***	19 (21.8%)	19 (28.4%)	18 (34.0%)	35 (34.0%)
Had visit where no contraception was due or given	NA***	47 (54.0%)	41 (61.2%)	31 (58.5%)	68 (66.0%)
TFV-DP adherence measures****					
Result available (N)	117	103	70	43	–
4 ≤ pills/week (% of total)	29 (17.7%)	37 (22.6%)	31 (18.9%)	20 (12.2%)	–
4 ≤ pills/week (% of tested)	29 (24.8%)	37 (35.9%)	31 (44.3%)	20 (46.5%)	–
Median TFV-DP levels (IQR)	492 (353–697)	494 (115–839)	637.5 (41.6–888)	590 (38.9–1032)	528.5 (133–821)

*With a 2-week window period for month 1 and 2 visits, and thereafter a 1-month window period for 2-month schedule and a 1.5 month window period for 3-month schedule

**Exit date defined as last visit date with no subsequent visits

***Injectable contraception was not typically due when the participant returned for month 1 bloods, but would have been due at subsequent visits

****Cut-off of ≥ 700 fmol/punch to approximate 4 ≤ pills/week

There were 103 participants on injectable or oral contraception at baseline who attended any follow-up visits (33 were on other types of contraception, and 28 did not attend follow-up visits). These participants were assigned either 2- or 3-monthly PrEP visit schedules, intended to synchronise with their contraception due dates, and 82.5% received their initial contraception type at one or more visit. However, 68 (66.0%) had a visit where no contraception was given, because it was not due, and 35 (34.0%) received another type of contraception (excluding condoms) at one or more of their follow-up visits. Of these, 25 were initially receiving the bi-monthly norethisterone enantate, and instead received the three-monthly medroxyprogesterone acetate injectable (Table 2).

**Fig. 1:**
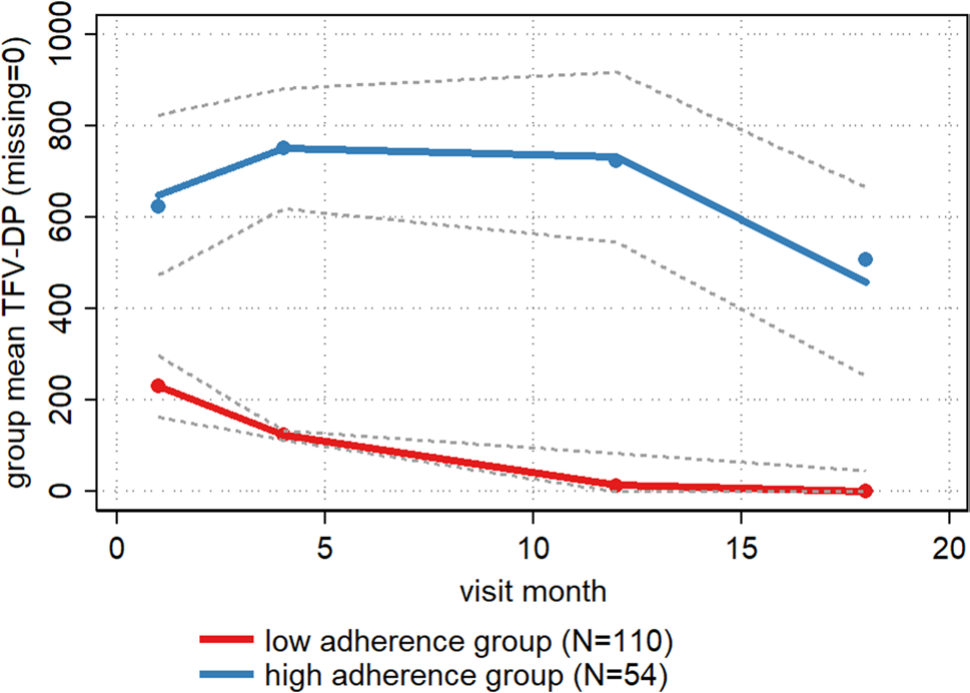
Plot of average TFV-DP levels over time by latent group identified in trajectory analysis

Of all participants, including those not tested, 29 (17.7%) had TFV-DP levels corresponding to 4 ≤ pills/week (≥ 700 fmol/punch) at their month 1 visit, declining to 20 (12.2%) by their 18-month visit. As a proportion of those who attended and had TFV-DP tests completed, TFV-DP levels corresponding to 4 ≤ pills/week (≥ 700 fmol/punch) was 24.8% at month 1, 35.9% at month 4, 44.3% at month 12 and 46.5% at month 18 (Table 2 and Fig. 1).

### Adherence Trajectories

Participants could be grouped into two trajectories of adherence (Fig. 1). Group 1 represented 110 (67%) of the study sample and had low adherence throughout, with median TFV-DP levels of 86 (IQR 0–398), declining to 0 (IQR 0–0) at months 12 and 18. In the second group (“high adherence group”, N = 54), median levels were 600.5 (IQR 443–831) at month 1, 758.5 (IQR 494–965) at month 3/4, 777 (IQR: 431–1002) at month 12, and 313.5 (IQR 0–844) at month 18 (see Supplementary Tables 1 and 2 for fit statistics and all group means and medians). Group 1 showed less variation than Group 2 (see Supplementary Fig. 1 for individual trajectories).

### Predictors of Adherence and Persistence

Few baseline characteristics predicted TFV-DP trajectory group or visit attendance. Being on injectable contraception or the oral pill, which requires regular clinic attendance, showed no protective effect against becoming lost to follow-up (HR: 1.1; 95% CI 0.7–2.0) or not completing the study (HR: 1.3; 95% CI 0.8–2.1). Participants with only primary school education were slightly less likely to return for PrEP initiation (RD: − 20.3, 95% CI − 39.4 to − 1.1), but among those who initiate, those that have tertiary education are more likely to become lost to follow-up (HR: 2.3, 95% CI 1.2–4.2) or not complete the study (HR: 1.8, 95% CI 1.0–3.1). See Supplementary Table 3 for all risk differences and hazards ratios.

The qualitative data did not link specific individual characteristics with persistence or adherence. Most participants described wanting additional protection from HIV and feeling strongly motivated to use PrEP, but this did not translate to consistent PrEP use. Participants spoke about the work and challenges that they faced in attempting to integrate daily PrEP into their lives. This process of “working it out” involved making decisions about (1) who to inform about their PrEP use and why, (2) dealing with side effects and (3) clinic attendance and clinic procedures. It is the inability to reconcile their PrEP use with one or more of these factors that led to missed doses or withdrawal from the PrEP program completely.

### Participant Experiences, Risk Perception and Behaviours Over Time

Among women who had at least one visit in each time period (n = 58), self-reported sex with more than one partner decreased significantly after enrolment (p < 0.001). The proportion reporting sex without a condom decreased at every time interval (p = 0.063). In the first six months of follow-up, 16 women (27.6%) had a syndromic STI, decreasing to 8 (13.8%) in the final six months (Table 3, see Supplementary Table 4 for overall proportions). In the individual interviews, participants credited their PrEP experience with changing their behaviour, with one commenting that “*[the PrEP nurse and counsellor] also support me in many ways besides issues of PrEP”* [PrEP user, 25 years old]. Another participant, who reported multiple concurrent partners prior to PrEP, explained:

“…when I started with PrEP and meeting with the PrEP counsellor, I then realised that no this thing of having too many boyfriends and sleeping with them without a condom is not okay... PrEP staff really advise us on how we should look after ourselves and so on, and you also realise which are the good ways and which are the bad ways, because there are too many infections out there and STIs. And you can't say you got it from that person when it could be the other, and even then, you simply don't know that person. So, it's better to have one person so you know where you got an STI from, instead of having many partners to a point where you don’t know who infected you. So, it really taught me a lot.” [PrEP user, 26 years old]

**Table 3:** Self-reported risk behaviours and perception, STI diagnoses and reported barriers and facilitators to PrEP use, among participants with any available data for each period, N = 58

	Enrolment*	Month 1–6^‡^	Month 8/9–12^‡^	Month 14/15–18^‡^	Kendall's tau-b^§^	p-Value^§^
High self-reported risk	29 (50%)	21 (36.2%)	16 (27.6%)	15 (25.9%)	− 0.173	0.004
Sex with more than one sexual partner**	30 (51.7%)	10 (17.2%)	11 (19%)	12 (20.7%)	− 0.21	< 0.001
Sex without a condom?**	51 (87.9%)	50 (86.2%)	47 (81%)	44 (75.9%)	− 0.112	0.063
Sex with person of unknown status**^†^	41 (70.7%)	50 (86.2%)	48 (82.8%)	47 (81%)	0.071	0.240
Sex with known positive**	1 (1.7%)	4 (6.9%)	3 (5.2%)	3 (5.2%)	0.041	0.492
STI (syndromic)	6 (10.3%)	16 (27.6%)	7 (12.1%)	8 (13.8%)	− 0.014	0.811
Reported barrier						
Negative reaction from						
Family	N/A	2 (3.4%)	0 (0%)	0 (0%)	− 0.125	0.084
Friends	N/A	1 (1.7%)	0 (0%)	0 (0%)	− 0.088	0.225
Partner	N/A	0 (0%)	0 (0%)	0 (0%)	–	1
Other prep users	N/A	0 (0%)	0 (0%)	0 (0%)	–	1
Forgetting to take pill	N/A	46 (79.3%)	38 (65.5%)	35 (60.3%)	− 0.157	0.029
Pill left at home	N/A	14 (24.1%)	4 (6.9%)	2 (3.4%)	− 0.25	0.001
Experienced side effects	N/A	4 (6.9%)	0 (0%)	0 (0%)	− 0.177	0.014
Reported facilitator						
Encouragement from						
Family	N/A	54 (93.1%)	51 (87.9%)	45 (77.6%)	− 0.173	0.016
Friends	N/A	45 (77.6%)	33 (56.9%)	24 (41.4%)	− 0.283	< 0.001
Partner	N/A	36 (62.1%)	27 (46.6%)	27 (46.6%)	− 0.12	0.096
Other prep users	N/A	52 (89.7%)	53 (91.4%)	43 (74.1%)	− 0.168	0.020
Whatsapp reminders	N/A	58 (100%)	58 (100%)	58 (100%)	–	1

*STI at screening

**“in the past 6 months” at enrolment, else ‘since last visit’

^‡^Reported at any visit during period, with a 2-week window period for month 1 visits, and thereafter a 1-month window period for 2-month schedule and a 1.5 month window period for 3-month schedule

^†^Defined as participant not seeing proof of partner’s negative test in three months prior to sex

^§^p-Value for Kendall’s rank coefficient test

The non-judgmental way that advice was delivered was also highlighted:

“They advise me and show me on what's right and wrong, and the consequences of naughty behaviour. They have an education approach that doesn't make me scared. They encourage me in a way that makes me correct my mistakes.” [PrEP user, 22 years old]

The main reported barrier to taking PrEP was forgetting to take or travel with the pills, with few reporting discouragement from friends, partners or family. Social support as a facilitator to PrEP adherence was reported more frequently at six months compared to 18 months. This included encouragement from family (93.1% vs 77.6%, p = 0.016), friends (77.6% vs 41.4%, p ≤ 0.001), partners (62.1% vs 46.6%, p = 0.096), and other PrEP users (89.7% vs 74.1%, p = 0.020). Whatsapp reminders were reported to be helpful throughout (Table 3). Among women who attended both month 1 and 6 visits (n = 64), social support as a reported facilitator increased between month 1 and 6 (Supplementary Table 5).

In the qualitative interviews, participants also reported disclosing their PrEP to family, partners, and friends, though disclosure depended on the type of relationship that the PrEP user had with the friend or family member, whether the PrEP user anticipated a positive reaction from the person they were disclosing to, and whether a negative response would adversely affect the participant. Reactions to participants’ disclosures were generally positive. In some cases, PrEP disclosure prompted conversations and peer education: “*I don't hide it. I just take my pills and when they ask what it is, I tell them it's PrEP and tell them how it works until they understand*” [PrEP user, 26 years old]. Most participants hoped that friends would follow their example or actively encouraged their friends to initiate PrEP, sometimes successfully. One mother of a participant reported that her daughter educated her about termination of pregnancy when she had an unplanned pregnancy, and another reported more open discussion around sex:

Interviewer: “Are you now doing things differently in your relationship compared to before? Say, in the way you talk about sex and condom use?

Interviewee: There is a difference. She used to fear me. But now, since...things have changed, she's now open. I sit her down and tell her it won't help her to talk to her friends about her problems. She must be my friend.” [Aunt of participant, 50 years old]

Support from other PrEP users was also mentioned in individual interviews, as was the participant Whatsapp group, which was very active throughout the study period. Despite the fact that clubs never proved feasible, two interviewees suggested group counselling sessions when asked how counselling could be improved. For those who attended the participant events, feedback was positive about the added value of sharing experiences with peers.

“sometimes there's something that you're scared to share or you think it happens to you only, but then we are together there as girls, you realise that your problem is better or realise that it also happens to other people as well. So you hear other stories, and that's what makes it to be nice. Because there are things that you can't talk about with PrEP staff but as a group you laugh and talk and we are not judging each other.” [PrEP user, 26 years old]

Although six interviewed PrEP users identified family planning as their point of entry for the PrEP study, only one mentioned integration of family planning as a facilitator to PrEP use. When asked generally about the advantage of getting PrEP at the study clinic, she stated *“they have made my life easy…I am able to come to PrEP on my appointment date for family planning”* [PrEP user, 24 years old]. Six others highlighted the importance of their geographic proximity to the clinic, often highlighting transport: *“It's fine with me since I don't have to use any transport to get here”* [PrEP user, 21 years old]. A 20-year old PrEP user stated *“I walk to the clinic. I do not have to use transport to the clinic so it is easy to get to it”*. Asked about going to another clinic for PrEP, a 24-year old PrEP user stated: *“This clinic is close. I won't lie and say I'd continue. I'd be lazy because other clinics are far”*.

At the first participant event, participants were asked about ways to make SRH clubs feasible, and while most expressed interest in clubs, they were unable to commit to times because of the need to prioritize unpredictable work and study schedules, or unplanned events.

## Discussion

We have presented characteristics, experiences, and outcomes of a cohort of 164 young women who initiated daily oral PrEP in conjunction with SRH services. The study occurred in a primary healthcare setting, in a DoH-run clinic, with no financial compensation for attendance. Our results are in line with other studies showing intermittent engagement, and a large proportion of PrEP discontinuations (48.2%) occurring within the first six months [19, 20, 40, 44]. Although many women did not sustain PrEP use, a minority continued to engage in PrEP services, and a significant proportion showed sustained good adherence. No measured baseline characteristics were strong predictors of persistence in PrEP use or visit attendance, supporting qualitative data that a constantly changing set of individual circumstances and priorities determine participants’ ability to persist with PrEP. One participant, who was likely in the window period at enrolment, tested positive for HIV in the study.

In a latent trajectory analysis of TFV-DP levels, a third of women fell into a group that showed, on average, high adherence throughout, with some drop-off at month 18. The remaining 110 participants showed low average adherence throughout, with the majority having no detectable (or tested) TFV-DP levels at each visit after month 1. This is an interesting contrast to a recent analysis of data from the 3Ps for Prevention Study (3P) [40] in a similar population, where PrEP was also given monthly for the first 3 months, followed by longer visit intervals. Their study showed two latent groups (48%, 52%), both of which had higher early TFV-DP levels than our respective ‘low’ and ‘high’ groups. However, in that study, both groups rapidly declined to very low adherence levels by month 12. There are several potential explanations for these different observed trajectories. Firstly, the 3P study reimbursed participants for attendance, which may have improved initial attendance, but selected for women who were less self-motivated to continue PrEP when visits were less frequent. Secondly, for the first three months, participants were told their TFV-DP results, and some were given additional incentives, conditional on high TFV-DP levels, which was associated with higher adherence [44]. While these effects are interesting, and incentivizing good adherence may be an important tool in some contexts, our results suggest that a significant minority of women have high adherence without such incentives, and this adherence is more sustained. Even in low resource settings, as a known effective HIV prevention strategy, PrEP should be offered as part of the standard SRH package, if only for this motivated minority.

Moreover, the inclusion of PrEP into an SRH package has positive effects beyond individuals’ PrEP use. PrEP provided an additional entry point for engagement in SRH care, and repeated visits provided an opportunity to administer hormonal contraception and counsel young women on risk reduction. Our qualitative and quantitative data suggests that this had an effect on behaviour, with decreasing risk behaviours reported over time, attributed by participants to the risk-reduction counselling. Relationships built with other PrEP users, through virtual platforms or physical meetings, could also provide long-term social support. A high proportion of participants reported that PrEP use was facilitated by support from friends and family, in line with qualitative research done in three sub-Saharan contexts where youth perceived peer influence and social support as potential facilitators to PrEP uptake [45]. This also suggests that most participants told at least some friends and family members about PrEP. In some cases this helped open dialogue around sex, and helped to educate others about PrEP. This echoes findings from HPTN 082 trial participants in similar contexts, where participants reported disclosure (when they had the right information and support to do so) as an empowering experience and an opportunity to educate and influence others [46]. Increasing awareness of PrEP as part of a broader package of sexual and reproductive health can also help destigmatize PrEP by framing it as something anyone can opt into at an appropriate time [47].

Recognising the challenges with, but overall benefits from a package of care that includes the option of PrEP, lessons drawn from this study can help maximise persistence on PrEP within resource constraints. PrEP providers need to address participants’ need for both convenience and social support, persistent themes in the quantitative and qualitative data that we discuss below.

To be sustainable, women need to be able to conveniently access PrEP and find ways to integrate it into their daily routines and other health needs. This was evidenced by the fact that the main reported barrier to taking PrEP was forgetting to take or travel with the pills. The unpredictability of their schedules was given as a reason for women not being able to attend the envisioned “SRH clubs”, which likely explains frequent lateness for scheduled visits too. Geographic proximity was also important: despite advertising the study at another clinic in Khayelitsha, and on the radio, most participants who enrolled were those who attended the clinic already, and aside from loss to follow-up and study completion, the main reason for study exit was the participant leaving the area. In the individual interviews, PrEP users expressed a strong preference against travelling further to receive their PrEP. More widespread availability of PrEP would help address these issues, including innovations such as pharmacy-led PrEP delivery [48]. To maximize participant convenience, we attempted to align contraception and PrEP visits for women on the pill or injectable contraception. However, women were often screened on a contraception date, and started PrEP within a month after screening, offsetting this schedule. This may also explain why we failed to see any relationship between contraception type and PrEP persistence. The current South African guidelines circumvent this issue, by allowing PrEP initiation on the day of initial screening and requiring participants to be called back if laboratory results are abnormal [49], as this is rare in this group [50]. Synchronizing of schedules was also complicated by changes in contraception methods, late or missed visits, and national shortages of the bi-monthly injectable norethisterone enantate [51]. PrEP provision needs to be adaptive to changing schedules to minimize unnecessary facility visits and gaps in PrEP possession.

Psycho-social support was also valued by participants. Encouragement from others, including support from other PrEP users, was reported as a facilitator to PrEP use increasingly over the first six months, but with declining frequency from 6 to 18 months. The high proportion (91%) who reported benefitting from support of other PrEP users, despite the fact that fewer than a third ever attended an organised participant event, may reflect previously existing networks, and the peer support offered over the Whatsapp group. Ideally, the option of connecting to peer support, through in-person group counselling, group gatherings, or virtual platforms, should be flexibly available to PrEP users when they need it most, likely in the first six months of PrEP use. A recent systematic review found a paucity of evidence for PrEP support via electronic and other new media technology interventions, with no studies looking at the outcomes of PrEP adherence and persistence [52], so this is potentially an area for further study. Our experience suggests that social support can be best provided through whichever platform is already widely used in the context (in our case, Whatsapp). Study counselling likely prepared participants to be open about PrEP use with friends, family, and partners, as they were knowledgeable enough to answer questions about PrEP, and combat any misconceptions. This highlights the importance of strong initial counselling and education, and community education, as it potentially has the indirect effect of facilitating openness about PrEP use and increasing social support.

The promise of long-acting PrEP [53, 54] will help to address some, but not all, of the challenges described above. PrEP users will no longer have the burden of daily pills, and the disclosure process that this sometimes necessitates. However, counselling around disclosure should still be a consideration in PrEP programs, given the benefits of social support, and peer and family education. Sustained attendance was a greater challenge to our program than adherence among those who did attend, and attendance is also important for long-acting methods such as three-monthly injectable PrEP. While we strongly support the development of more convenient, and long-acting methods, the lessons outlined above will remain relevant. Programs need to continue to balance participants’ need for both convenience and psycho-social support.

Our study had several limitations. Firstly, risk behaviour was self-reported, and therefore subject to desirability bias. However, our staff created good rapport with participants, and in a similar context, PrEP study participants reported few barriers to disclosing risk behaviours to study staff (57% reporting no barriers) [55]. It may have been the case that participants became more likely to report true behaviours over the study period, but if this were the case then the observed decline in risk behaviours would be an under-estimate of effect. Secondly, participant event attendance was under-ascertained because of a missing register, but this accounted for just one of the seven events. Thirdly, our latent group adherence trajectory analysis identified two distinct groups, but it is possible, in a larger sample, that more groups could have been identified by through this method. It is also possible that the assumption of low adherence (TFV-DP = 0) among those not attending the relevant visits is inaccurate, but we can assume this means a gap in PrEP use at some point around this period, as participants were unlikely to receive PrEP anywhere outside the study context. If this assumption is incorrect, it would not change the key finding that a core group of women showed, on average, good adherence throughout: if anything the average TFV-DP levels in this group would be higher. In addition, adherence levels, measured by TFV-DP, are only estimates of true pill-taking, as there is variation in metabolisation of TFV between people, and over time [56, 57]. Fourthly, our results should be generalised with caution to environments that are less ‘youth-friendly’, as our study site was a youth clinic and our study staff were trained and selected to engage with young women in a non-judgmental way. Our study participants, enrolled in an era when PrEP was still relatively unknown, might have been particularly confident and motivated women. However, this is unlikely to invalidate the lessons we have described above regarding the need for convenience and social support. Finally, this study was not powered for a full multivariate analysis of predictors of PrEP adherence or persistence. As such, our quantitative data does not definitively rule out the possibility that there are more statistically significant baseline predictors of good adherence. However, these findings should be considered in the context of all the data and experiences presented indicating that these determinants are complex and multifaceted.

## Conclusions

Integrating PrEP into SRH care provides an additional entry point for engagement and has positive effects beyond PrEP use, leading to increased engagement with SRH care, peer education, and possibly a reduction in risk behaviours. More widely and conveniently available PrEP would assist young women to persist on PrEP, but psycho-social support was also a facilitator to PrEP use, especially in the first few months. Such support can be integrated into programs through counselling, platforms such as Whatsapp, formal or informal PrEP user gatherings, and equipping PrEP users to disclose to existing social networks where appropriate. Our findings highlight that integrating PrEP into SRH services for young women in low-income settings is valuable, but needs to be flexible enough to provide convenience and support, as needed, within resource constraints.

## Supplementary Information

Below is the link to the electronic supplementary material.

Supplementary file1 (DOCX 85 kb)

## Data Availability

An anonymised dataset is made available on request per MSF’s data sharing policy (https://fieldresearch.msf.org/handle/10144/306488). For access to the data, please email data.sharing@msf.org.

## Code Availability

Stata code for all analyses are available here: https://github.com/talicassidy/prep_analysis
