# Supplementary material for: Delivering PrEP to Young Women in a Low-Income Setting in South Africa: Lessons for Providing Both Convenience and Support
Source: AIDS Behav. 2021 Jul 14;26(1):147–59. doi: 10.1007/s10461-021-03366-x (PMC8786762; doi:10.1007/s10461-021-03366-x)
Supplement: Supplementary file 1 — Supplementary file1 (DOCX 85 kb) [file 10461_2021_3366_MOESM1_ESM.docx]

# Supplementary Figures and Tables

Supplementary table 1 Model fit statistics for different shape parameters, with missing TFV-DP levels coded as zero

| Shape parameters* | K | BIC (N=656) | BIC (N=164) | AIC | log likelihood | smallest group size | average posterior probability per group | | |
| --- | --- | --- | --- | --- | --- | --- | --- | --- | --- |
| *2, 2* | *2* | *-2505.31* | *-2499.76* | *-2487.36* | *-2479.36* | *33%* | *97%* | *96%* |  |
| 1, 1 | 2 | -2502.79 | -2498.63 | -2489.33 | -2483.33 | 33% | 97% | 96% |  |
| 1, 2 | 2 | -2502.1 | -2497.24 | -2486.39 | -2479.39 | 33% | 97% | 96% |  |
| *2, 2, 2* | 3 | -2486.44 | -2478.12 | -2459.52 | -2447.52 | 15% | 94% | 95% | 97% |
| 1, 2, 2 | 3 | -2483.82 | -2476.19 | -2459.15 | -2448.15 | 15% | 94% | 94% | 97% |
| 1, 1, 2 | 3 | -2480.08 | -2473.15 | -2457.65 | -2447.65 | 15% | 95% | 91% | 97% |

*Models with three or more groups did not converge

Supplementary figure 1 Participant TFV-DP levels over study period by 2 trajectory groups

Supplementary table 2 Median and mean TDF-DP (fmol/punch) levels for months 1, 3/4, 12 and 18

|  | Group 1 | Group 2 | Total |
| --- | --- | --- | --- |
| N | 110 | 54 | 164 |
| Month 1 median (IQR) | 86 (0-398) | 600.5 (443-831) | 366 (0-628.5) |
| Month 1 mean | 236.4282 | 617.2278 | 361.8134 |
| Month 3/4 median (IQR) | 0 (0-115) | 758.5 (494-965) | 65.15 (0-644) |
| Month 3/4 mean | 129.7136 | 752.1704 | 334.6689 |
| Month 12 median (IQR) | 0 (0-0) | 777 (431-1002) | 0 (0-416) |
| Month 12 mean | 6.782727 | 747.6537 | 250.728 |
| Month 18 median (IQR) | 0 (0-0) | 313.5 (0-844) | 0 (0-0) |
| Month 18 mean | 0 | 517 | 170 |

Supplementary table 3 Risk difference associating baseline characteristics with risk of enrolment (n=164, N=236) and risk being in the high adherence trajectory group (n=54, N=164); Hazards ratios for baseline characteristics and rate of LTFU (n=78, N=164) and rate of any study exit before completion (n=117, N=164)

|  | **% risk difference(95% CI)** | | **HR (95% CI)** | |  |
| --- | --- | --- | --- | --- | --- |
| **Dependent variable:** | **Enrolment** | **Group 2: high adherence group** | **LTFU** | **Study incomplete** | |
| Recruited by nurse | -19.7 (-89.2-49.9) | - | - | 1.6 (0.2-11.7) | |
| Recruited by clinic counselor | 8.8 (-3.5-21.2) | 4.3 (-11.2-19.9) | 1.0 (0.6-1.7) | 1.0 (0.7-1.5) | |
| recruited by study staff | -8.1 (-20.6-4.4) | 6.1 (-9.4-21.6) | 0.7 (0.4-1.1) | 0.7 (0.5-1.1) | |
| Recruited by outreach outside of clinic | -20 (-60.4-20.4) | 0.4 (-53.4-54.3) | 0.8 (0.1-5.6) | 1.0 (0.3-4.2) | |
| Recruited by word of mouth | **17.5 (4.2-30.9)** | -9.8 (-26.9-7.2) | 1.6 (0.9-2.7) | 1.4 (0.9-2.2) | |
| Recruited by poster or flier in clinic | -16.3 (-35.4-2.8) | -1.9 (-25.8-22.1) | 1.0 (0.5-2.2) | 1.2 (0.7-2.2) | |
| Recruited by other | 2.0 (-32.0-36.0) | -13.3 (-49.2-22.5) | 1.4 (0.5-4.6) | 1.0 (0.3-3.0) | |
| Completed primary school | **-20.3 (-39.4- -1.1)** | 7.8 (-18.1-33.7) | 1.0 (0.5-2.0) | 0.9 (0.5-1.6) | |
| Completed secondary/high school | **14.7 (0.2-29.3)** | 6 (-11.6-23.5) | 0.6 (0.4-1.1) | 0.8 (0.5-1.2) | |
| Completed tertiary education | -4.6 (-23.9-14.7) | -17 (-36.7-2.6) | **2.3 (1.2-4.2)** | **1.8 (1.0-3.1)** | |
| employed | 4.0 (-10.7-18.7) | -2.1 (-20-15.9) | 1.1 (0.6-2.0) | 1.2 (0.8-1.9) | |
| Studying | 1.4 (-10.7-13.4) | 2.4 (-12.4-17.2) | 0.9 (0.6-1.4) | 0.9 (0.6-1.3) | |
| Employed and studying | -16.6 (-44.3-11.2) | 10.4 (-27-47.8) | 1.3 (0.5-3.6) | 1.1 (0.4-2.6) | |
| none of the above | -0.8 (-13-11.4) | -4.6 (-19.4-10.2) | 1.1 (0.7-1.7) | 1.0 (0.7-1.5) | |
| Stayed in Khayelitsha less than a year | 10.3 (-9.1-29.6) | 7.8 (-18.1-33.7) | 1.2 (0.6-2.4) | 0.9 (0.5-1.8) | |
| Stayed in Khayelitsha 1-3 years | -14 (-33.3-5.3) | -15.7 (-36.3-4.9) | 1.3 (0.6-2.5) | 1.3 (0.7-2.3) | |
| Stayed in Khayelitsha greater than 3 years | 4.4 (-10.6-19.4) | 4.8 (-13.1-22.7) | 0.8 (0.5-1.4) | 0.9 (0.6-1.4) | |
| Age (in years) | -1.6 (-4.6-1.3) | -0.7 (-4.4-3.1) | 0.9 (0.8-1.1) | 1.0 (0.9-1.1) | |
| Sexually active | -5.6 (-48.5-37.2) | -0.4 (-54.3-53.4) | - | 1.1 (0.3-4.3) | |
| STI diagnosed at screening | -4.5 (-22.9-13.9) | -13.4 (-33.3-6.5) | 0.9 (0.4-1.9) | 1.2 (0.7-2.0) | |
| PEP started at screening | 3.6 (-16.0-23.2) | 5.1 (-19.8-30) | 0.9 (0.4-1.9) | 0.8 (0.4-1.5) | |
| high self-reported risk perception | N/A | -2.4 (-16.8-11.9) | 1.4 (0.9-2.3) | 1.2 (0.8-1.7) | |
| sex with more than one partner in past six months | N/A | 2.9 (-11.8-17.5) | 0.7 (0.5-1.2) | 0.7 (0.5-1.1) | |
| sex without a condom in past six months | N/A | -15.1 (-41.4-11.2) | 2.3 (0.8-6.2) | 1.6 (0.8-3.3) | |
| sex without person of unknown status in past six months | N/A | 2.3 (-13.2-17.7) | 1.0 (0.6-1.7) | 1.0 (0.7-1.5) | |
| on injectable contraception/pill | N/A | -11.9 (-30.5-6.7) | 1.1 (0.7-2.0) | 1.3 (0.8-2.1) | |
| Baseline contraception: 2-monthly injectable | N/A | 5.3 (-10.4-20.9) | 1.1 (0.7-1.8) | 1.0 (0.7-1.5) | |
| Baseline contraception: 3-monthly injectable | N/A | -12.6 (-26.8-1.6) | 1.1 (0.7-1.7) | 1.3 (0.9-1.8) | |
| Baseline contraception: IUD/implant | N/A | 14.5 (-7.7-36.6) | 0.7 (0.4-1.5) | 0.6 (0.3-1.1) | |
| Baseline contraception: oral pill | N/A | 0.4 (-31.2-32.1) | 0.6 (0.2-2.0) | 0.7 (0.3-1.7) | |
| Baseline contraception: condoms | N/A | 7.5 (-23.7-38.8) | 1.1 (0.4-2.7) | 1.2 (0.6-2.4) | |

Supplementary table 4 Reported risk behaviours and barriers and facilitators to PrEP use among all participants (N=164)

| N=164 | Enrolment* | month 1-6^‡^ | month 7-12^‡^ | month 13-1^‡^8 |
| --- | --- | --- | --- | --- |
| high self-reported risk | 82 (50.0%) | 57 (34.8%) | 26 (15.9%) | 20 (12.2%) |
| sex with more than one sexual partner** | 69 (42.1%) | 19 (11.6%) | 14 (8.5%) | 17 (10.4%) |
| sex without a condom? ** | 149 (90.9%) | 102 (62.2%) | 64 (39.0%) | 50 (30.5%) |
| sex with person of unknown status | 5 (3.0%) | 12 (7.3%) | 5 (3.0%) | 3 (1.8%) |
| sex with known positive** | 19 (11.6%) | 27 (16.5%) | 8 (4.9%) | 9 (5.5%) |
| STI (syndromic) | 82 (50.0%) | 57 (34.8%) | 26 (15.9%) | 20 (12.2%) |
| Reported barrier:  negative reaction from: |  |  |  |  |
| family | N/A | 3 (1.8%) | 1 (0.6%) | 0 (0.0%) |
| friends | N/A | 1 (0.6%) | 0 (0.0%) | 0 (0.0%) |
| partner | N/A | 1 (0.6%) | 0 (0.0%) | 0 (0.0%) |
| other PrEP users | N/A | 0 (0.0%) | 0 (0.0%) | 0 (0.0%) |
| Forgetting to take pill | N/A | 87 (53.0%) | 52 (31.7%) | 37 (22.6%) |
| Pill left at home | N/A | 26 (15.9%) | 4 (2.4%) | 3 (1.8%) |
| Experienced side effects | N/A | 15 (9.1%) | 0 (0.0%) | 0 (0.0%) |
| Reported facilitator:  encouragement from: |  |  |  |  |
| family | N/A | 128 (78.0%) | 81 (49.4%) | 68 (41.5%) |
| friends | N/A | 111 (67.7%) | 71 (43.3%) | 53 (32.3%) |
| partner | N/A | 83 (50.6%) | 48 (29.3%) | 27 (16.5%) |
| other PrEP users | N/A | 63 (38.4%) | 36 (22.0%) | 30 (18.3%) |
| Whatsapp reminders | N/A | 105 (64.0%) | 71 (43.3%) | 49 (29.9%) |
| *STI at screening; **“in the past 6 months” at enrolment, else ‘since last visit’; ‡reported at any visit during period. ^‡^with a 1-month window period for 2-month schedule and a 1.5 month window period for 3-month schedule | | | | |

Supplementary table 5 Reported risk behaviours and barriers and facilitators to PrEP use among participants attend month 1 and 6 visits (N=64)

|  | Enrolment* | month 1^‡^ | month 6^‡^ |  |
| --- | --- | --- | --- | --- |
| high self-reported risk | 54.7% | 15.6% | 20.3% |  |
| sex with more than one sexual partner** | 43.8% | 4.7% | 7.8% |  |
| sex without a condom? ** | 92.2% | 5% | 65.6% |  |
|  |  |  |  |  |
| sex with known positive** | 3.1% | 1.6% | 4.7% |  |
| STI (syndromic) | 9.4% | 3.1% | 7.8% |  |
| Reported barrier:  negative reaction from: |  |  |  |  |
| family | N/A | 1.6% | 1.6% |  |
| friends | N/A | 0.0% | 0.0% |  |
| partner | N/A | 0.0% | 0.0% |  |
| other PrEP users | N/A | 0.0% | 0.0% |  |
| Forgetting to take pill | N/A | 46.9% | 42.2% |  |
| Pill left at home | N/A | 6.3% | 3.1% |  |
| Experienced side effects | N/A | 4.7% | 0.0% |  |
| Reported facilitator:  encouragement from: |  |  |  |  |
| family | N/A | 65.6% | 78.1% |  |
| friends | N/A | 40.6% | 50.0% |  |
| partner | N/A | 25.0% | 37.5% |  |
| other PrEP users | N/A | 54.7% | 76.6% |  |
| Whatsapp reminders | N/A | 82.8% | 95.3% |  |
| *STI at screening; **“in the past 6 months” at enrolment, else ‘since last visit’; ^‡^with a 1-month window period for 2-month schedule and a 1.5 month window period for 3-month schedule | | | | |
